# Comparative transcriptome analyses reveal the genetic basis underlying the immune function of three amphibians’ skin

**DOI:** 10.1371/journal.pone.0190023

**Published:** 2017-12-21

**Authors:** Wenqiao Fan, Yusong Jiang, Meixia Zhang, Donglin Yang, Zhongzhu Chen, Hanchang Sun, Xuelian Lan, Fan Yan, Jingming Xu, Wanan Yuan

**Affiliations:** 1 Chongqing Research Center of Conservation and Development on Rare and Endangered Aquatic Resources, Chongqing University of Arts and Sciences, Yongchuan, Chongqing, China; 2 Chongqing Key Laboratory of Kinase Modulators as Innovative Medicine, Yongchuan, Chongqing, China; 3 Chongqing Engineering Laboratory of Targeted and Innovative Therapeutics, Yongchuan, Chongqing, China; The National Orchid Conservation Center of China; The Orchid Conservation & Research Center of Shenzhen, CHINA

## Abstract

Skin as the first barrier against external invasions plays an essential role for the survival of amphibians on land. Understanding the genetic basis of skin function is significant in revealing the mechanisms underlying immunity of amphibians. In this study, we *de novo* sequenced and comparatively analyzed skin transcriptomes from three different amphibian species, *Andrias davidianus*, *Bufo gargarizans*, and *Rana nigromaculata* Hallowell. Functional classification of unigenes in each amphibian showed high accordance, with the most represented GO terms and KEGG pathways related to basic biological processes, such as binding and metabolism and immune system. As for the unigenes, GO and KEGG distributions of conserved orthologs in each species were similar, with the predominantly enriched pathways including RNA polymerase, nucleotide metabolism, and defense. The positively selected orthologs in each amphibian were also similar, which were primarily involved in stimulus response, cell metabolic, membrane, and catalytic activity. Furthermore, a total of 50 antimicrobial peptides from 26 different categories were identified in the three amphibians, and one of these showed high efficiency in inhibiting the growth of different bacteria. Our understanding of innate immune function of amphibian skin has increased basis on the immune-related unigenes, pathways, and antimicrobial peptides in amphibians.

## Introduction

Amphibians represent the transitional vertebrate taxon from aquatic to terrestrial life [1] and therefore also a long history of evolution at both molecular and phenotype levels before adaptation to the terrestrial environment. As the first defensive barrier, the skin of amphibians plays an essential role in protecting against external invasions [2]. However, for most amphibians, the skin is totally naked, making them highly susceptible to environmental factors and also the most threatened group of species on the planet [3]. Thus, understanding the molecular basis of skin functions may not only provide valuable information for amphibian conservation but also a foundation for studies on innate immunity.

Different from aquatic and terrestrial species, amphibian skin evolved versatile functions and complex structures to adapt to life on land [1]. First, skin is an important respiratory organ for amphibians, because the lungs of this taxon are not completely evolved [1, 4]. Moreover, amphibian skins evolved distinguishing structures from other species, such as the abundant glands in skin [5]. Mucous and granular glands are the two primary glands that form the adaptive functions of amphibian skins. The mucous glands secrete mucus, which retains skin moisture and protects against predators [6]. The granular glands are specialized reservoirs for antimicrobial peptides (AMPs), which are a variety of immunologically active substances used in defense against bacterial infections [7]. Thus, skin is an important contributor to the innate immune system of amphibians. Additionally, wound healing is promoted by some specific types of peptides that stimulate the expression of growth factors [8, 9], which makes amphibians a valuable natural medical resource.

Because of serious infectious diseases and environmental degradation, amphibian species are now globally imperiled and populations are decreasing [10, 11]. To better protect this taxon, several studies recently explored the molecular basis of the adaptive evolution and stress response in amphibians using “omics” methodology [1, 3, 7, 12, 13]. The newly developed *de novo* transcriptome sequencing technology is a powerful methodology for gene function identification, comparative genomics analyses, and evolutionary biology studies, particularly for non-model species and species with large genomes [14]. In this study, *de novo* transcriptome sequencing was performed for the skin from three different amphibian species, the Chinese giant salamander (*Andrias davidianus*), Asiatic toad (*Bufo gargarizans*), and Heiban frog (*Rana nigromaculata* Hallowell), which inhabit different environments and vary in skin phenotypes. With functional annotation and comparative analyses of these transcriptomes, we explored the genetic basis of immune functions of the skin of amphibians.

## Results

### Transcriptome assembly

The clean reads obtained after filter and quality control for *A*. *davidianus*, *B*. *gargarizans*, and *R*. *Hallowell* were 119,269,618, 117,350,510, and 111,152,846, respectively, which corresponded to clean bases of 17.82 G, 17.46 G, and 16.61 G, respectively (Table 1). The clean GC content of the three species ranged from 45.33 to 48.62%. A total of 167,064, 271,117, and 260,306 unigenes were generated for *A*. *davidianus*, *B*. *gargarizans*, and *R*. *Hallowell*, respectively, after *de novo* assembly and removal of the redundant sequences. The N50 lengths of unigenes for *A*. *davidianus*, *B*. *gargarizans*, and *R*. *Hallowell* were 956, 635, and 693 bp, respectively. Because all transcriptomes were *de novo* assembled by Trinity with default parameters, the N50 difference among them might result from the differences in genome sequence and structure among species. The length distributions of unigenes are shown in S1 Fig.

**Table 1:** Statistical summary of the transcriptome data and assembled unigenes of each amphibian skin library.

Species	*A*. *davidianus*	*B*. *gargarizans*	*R*. *Hallowell*
Clean reads	119,269,618	117,350,510	111,152,846
Clean bases	17,823,923,530	17,459,971,361	16,613,432,806
Clean GC%	48.62	46.78	45.33
Clean Q20%	98.89	98.8	98.82
Total number of unigenes	167,064	271,117	260,306
Total bases of unigenes	108,231,106	148,546,509	148,234,961
Mean length (bp)	647.84	547.91	569.46
Median length (bp)	360	343	349
N50 length (bp)	956	635	693

### Functional annotation of unigenes

The numbers and percentages of unigenes annotated in each database are shown in Table 2. In total, 56,692 (33.93%), 60,461 (22.30%), and 60,315 (23.17%) unigenes in *A*. *davidianus*, *B*. *gargarizans*, and *R*. *Hallowell* were annotated at least in one database. For the top NR hits on assembled unigenes, the species was the same for the three species, which was the model species *X*. *tropicalis* (S2 Fig), indicating that these transcriptomes were correctly assembled.

**Table 2:** Numbers and percentages of unigenes annotated in five different databases for each amphibian skin library.

Database	*A*. *davidianus*	*B*. *gargarizans*	*R*. *Hallowell*
Total Unigenes	167,064(100%)	271,117(100%)	260,306(100%)
COG	30,074(18.00%)	31,131(11.48%)	30,141(11.58%)
NR	52,868(31.65%)	56,043(20.67%)	55,906(21.48%)
Uniprot	39,222(23.48%)	41,773(15.41%)	42,536(16.34%)
GO	22,302(13.35%)	27,044(9.98%)	30,829(11.84%)
KEGG	23,256(13.92%)	23,716(8.75)	24,874(9.56%)
Annotated in all Databases	11,863(7.10%)	13,615(5.02%)	14,287(5.49%)
Annotated in at least one Database	56,692(33.93%)	60,461(22.30%)	60,315(23.17%)

**Fig 1:**
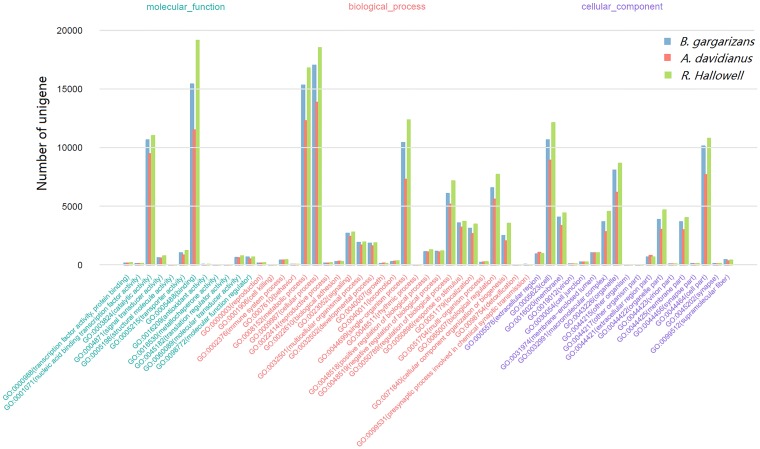
Histogram of GO annotation of the unigenes in each amphibian. The *x-axis* represents level two GO terms; the left *y-axis* represents unigene numbers in each GO term. Bars with different colors represent different species.

The GO functional classification of unigenes was performed using WEGO software[15]. The highly enriched GO terms were similar among the three amphibians (Fig 1). For the molecular function category, the most common GO terms were “catalytic activity” (GO:0003824) and “binding” (GO:0005488). For biological processes, the predominantly represented GO terms included “metabolic process” (GO:0008152), “cellular process” (GO:0009987), and “single-organism process” (GO:0044699). For the cellular component, the highly enriched GO terms were “cell” (GO:0005623), “organelle” (GO:0043226), and “cell part” (GO:0044464) (Fig 1). These results suggested that the skins of the three species shared similar functions. Unigenes in these terms were primarily involved in radical cellular activities. In addition to the basic functions, skins of amphibians play vital roles in immune response and anti-bacteria defense. In these areas, the GO terms of “immune system process” (GO:0002376) and “response to stimulus” (GO:0050896) also contained many unigenes.

**Fig 2:**
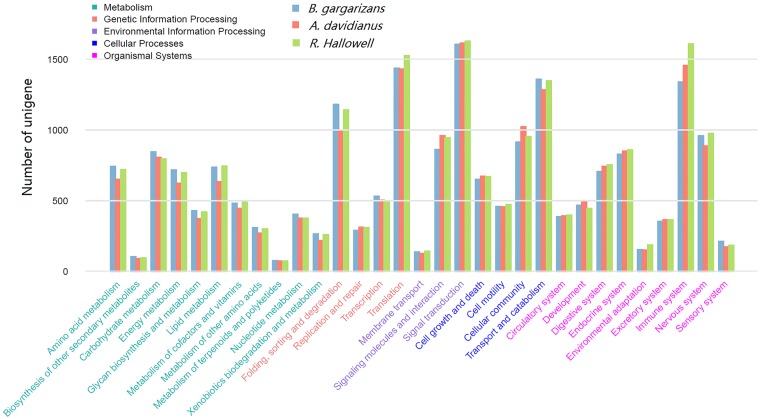
Histogram of KEGG pathway annotation of the unigenes in each amphibian. The *x-axis* represents hierarchy two pathways; the left *y-axis* represents unigene numbers in each pathway. Bars with different colors represent different species.

Additionally, KEGG pathway classification showed that the predominantly clustered hierarchy two pathways among the three amphibians were similar, which included “signal transduction”, “immune system”, “translation”, “transport and catabolism”, and “folding, sorting and degradation” (Fig 2). The large amount of unigenes in the “immune system” across the three amphibians were further indication that the skin plays an important role in immune function. The detailed KEGG pathway clusters and unigene numbers in each pathway are shown in S1 Table; the “ribosome” pathway (ko03010) contained the highest number of unigenes across the three amphibians.

### Evolutionary analysis based on transcriptome data

**Fig 3:**
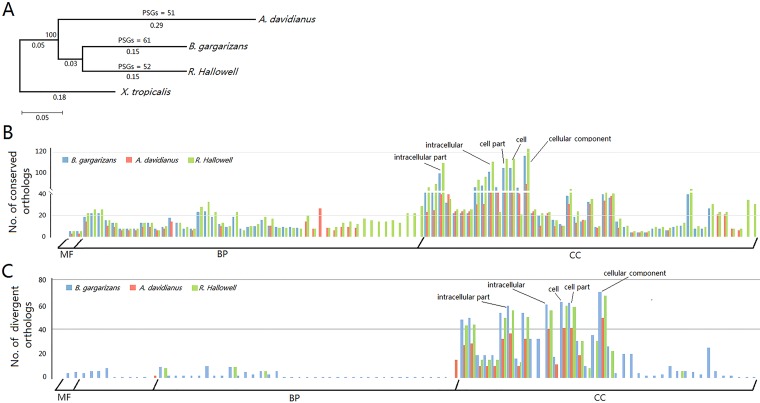
Comparative analyses of the three skin transcriptomes. (A) Phylogenetic tree of the three amphibians, *A*. *davidianus*, *B*. *gargarizans*, and *R*. *Hallowell*. Numbers under lines indicate evolutionary distance; the number above a line indicates bootstrap value. PSGs, positively selected genes. (B-C) Significantly enriched GO terms (FDR < 0.05) for conserved (B) and divergent (C) orthologs in each amphibian. Bars with different colors represent different species. MP, molecular function; BP, biological process; CC, cellular component.

A total of 8,844 ortholog groups were generated from the transcriptomes of the three species (*A*. *davidianus*, *B*. *gargarizans* and *R*. *Hallowell*). To analyze the phylogenetic relationships of the three amphibian species, a phylogenetic tree was constructed based on 1,147 single-copy orthologs using *X*. *tropicalis* as the out-group species (Fig 3A). The phylogenetic tree showed that *B*. *gargarizans* and *R*. *Hallowell* generated from a common ancestor species and were more closely related to *X*. *tropicalis* than to *A*. *davidianus*. This topology is consistent with an earlier study on the phylogeny of amphibians [16].

Additionally, Ka/Ks values of the 1,147 single-copy orthologs were calculated to evaluate the molecular evolution of the three amphibians. In total, 178, 198, and 202 conserved unigenes (Ka/Ks < 0.1) and 143, 155, and 142 divergent unigenes (Ka/Ks > 1) were identified in *A*. *davidianus*, *B*. *gargarizans*, and *R*. *Hallowell*, respectively (S3 Fig). GO classification (FDR < 0.05) for these conserved or divergent orthologs showed similar results across species, with the five most enriched GO terms “intercellular part,” “intracellular,” “cell part,” “cell,” and “cellular component” (Fig 3B and 3C; S2 Table). The large number of orthologs in the cellular component category suggested that the basic cell structures and cell components of these three species shared high similarity. By contrast, orthologs that clustered in the biological process category varied across the three amphibians, with the differences primarily represented in GO terms related to nucleotide metabolism, signal transduction, energy metabolism, and respiration (S2 Table). Variance of ortholog numbers in the biological process category provided evidence for functional differentiation among the skins of the three amphibians. KEGG enrichment (FDR < 0.05) revealed that the significantly clustered pathways for conserved orthologs in each amphibian were also similar and were primarily related to nucleotide metabolism and immune response (Table 3). However, no significantly enriched pathway was identified for divergent orthologs.

**Table 3:** Significantly enriched KEGG pathways for conserved orthologs in each amphibian.

ko ID	Pathway	*A*. *davidianus*		*B*. *gargarizans*		*R*. *Hallowell*	
		Orthologs (47)	*P*-adjusted	Orthologs (47)	*P*-adjusted	Orthologs (52)	*P*-adjusted
ko03020	RNA polymerase	4	0.002483226	4	0.005307277	4	0.003223486
ko00230	Purine metabolism	8	0.001224359	7	0.012332383		
ko05016	Huntington’s disease			8	0.012958128		
ko00240	Pyrimidine metabolism	8	4.0032E-05	5	0.012958128	7	0.000945342
ko04623	Cytosolic DNA-sensing pathway	3	0.044362395	3	0.023776887	4	0.004825662
ko00510	N-Glycan biosynthesis	4	0.003965558				

For positively selected genes (PSGs), 51, 61, and 52 PSGs were identified in *A*. *davidianus*, *B*. *gargarizans*, and *R*. *Hallowell*, respectively (S3 Table). Functional annotation of PSGs in each amphibian showed that they were primarily related to stimulus response, cell metabolic, membrane, and catalytic activity.

### Putative antimicrobial peptides (AMPs)

A total of 13, 13, and 24 putative AMPs were identified in *A*. *davidianus*, *B*. *gargarizans*, and *R*. *Hallowell*, respectively (Table 4), with nine that were common across these amphibians. The number of putative AMPs identified in *R*. *Hallowell* skin was almost twice the number in the skin of *A*. *davidianus* and *B*. *gargarizan*, and AMPs in the “macrotympanain”, “nigroain”, “OHTI precursor”, and “palustrin” families were specific to *R*. *Hallowell*. The AMP in the “ranacyclin Cc” family was specific to *A*. *davidianus* skin. However, no specific AMP was identified in *B*. *gargarizan* skin. Of the AMPs, “histone 2A” expressed to a high level in the skins of all three amphibians. The “Cathelicidin-OH antimicrobial peptide-like” and “Proteinase inhibitor PSKP-1” had significantly higher levels of expression in *A*. *davidianus* than in *B*. *gargarizans* and *R*. *Hallowell*. Of note, most of the AMPs identified in *R*. *Hallowell* showed high expression levels (Table 4). These results suggested that the three amphibian skins retained some common functions in pathogen resistance, while simultaneously evolving different mechanisms of resistance to specific pathogens to adapt to changes in environments.

**Table 4:** Categories and expression levels of putative AMPs in each amphibian skin.

Description	FPKM		
	*A*. *davidianus*	*B*. *gargarizans*	*R*. *Hallowell*
Amolopin-9LF1	0.85	NF	499.08
Andersonin-9 antimicrobial peptide precursor	NF^a^	4.92	7.23
Andersonin-U1	NF	NF	0.59
Brevinin-1Ed	13.84	13.75	8954.91
Brevinin-2Rc	NF	NF	9.47
Cathelicidin-OH antimicrobial peptide-like	1396.72	1.55	0.74
Esculentin-1A	30.81	12.71	17936.24
Esculentin-1a/b	NF	NF	0.94
Esculentin-2P	29.42	18.92	18173.85
Histone 2A	228.44	124.83	109.37
Liver-expressed antimicrobial peptide 2	2.4	0.69	4.55
Lividin-8	NF	1.4	1732.92
Macrotympanain-E1	NF	NF	18.92
Nigroain-A	NF	NF	456.92
Nigrocin-1	21.65	1.77	5760.43
Odorranain-C7 antimicrobial peptide precursor	NF	0.74	393.16
Odorranain-M1	1.53	NF	475.86
Odorranain-M2	NF	14.07	NF
Odorranain-P2a	NF	NF	0.64
OHTI precursor	NF	NF	3081.13
Palustrin-2GN1 antimicrobial peptide precursor	NF	NF	1.67
Palustrin-CU-A1	NF	NF	124.15
Pelophylaxin-2	8.97	0.79	10851.88
Proteinase inhibitor PSKP-1	6254.12	8.15	6.65
Ranacyclin Cc	5.75	6.74	NF
Skin peptide tyrosine-tyrosine	0.57	NF	1.74

^*a*^: not found.

### Antimicrobial assay with a putative AMP

**Fig 4:**
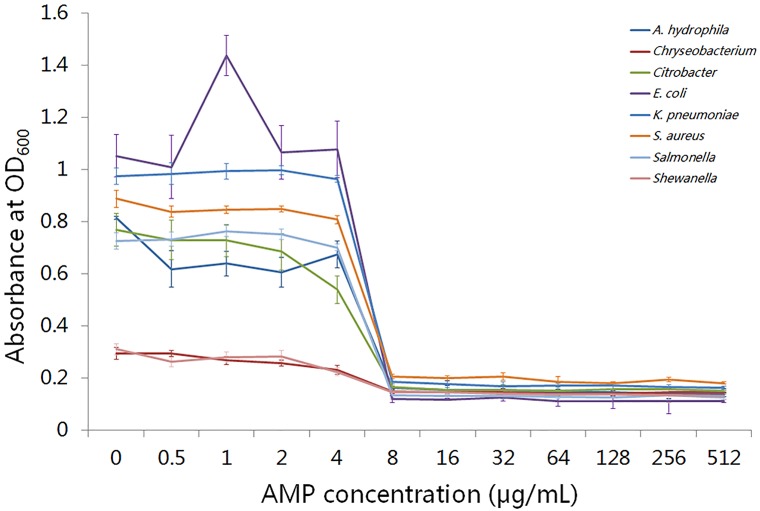
Minimum inhibitory concentration (MIC) assay of liver-expressed antimicrobial peptide 2. The *x-axis* represents concentration of liver-expressed antimicrobial peptide 2; the left *y-axis* represents absorbance values of bacteria cultures at OD_600_. The lines with different colors represent different bacteria.

To determine the antibacterial effect of putative AMPs, we performed an antimicrobial assay for a common putative AMP, “liver-expressed antimicrobial peptide 2,” across the three amphibians. As shown in Fig 4, liver-expressed antimicrobial peptide 2 completely inhibited the growth of eight different types of bacteria at the MIC value of 8 μg/mL. This result demonstrated that transcriptome sequencing was an effective way to identify AMPs in amphibian skin, which should facilitate further investigation of the immune functions of amphibian skin.

## Discussion

Amphibians are the transitional taxon in the evolutionary history of vertebrates. Thus, studies investigating the evolution of amphibians, particularly those that rely on molecular resources, are essential in linking the gap between aquatic and terrestrial species. However, the whole genome sequences for amphibians are now only available for the two model species *X*. *tropicalis* and *Xenopus laevis* (*X*. *laevis*) [17], which greatly limit the study of the evolution of this taxon. The newly developed *de novo* transcriptome sequencing technology, which directly targets functional genes, is a powerful method that can overcome this limitation in genome resources, particularly for non-model species [14]. Benefiting from this technology, several key genes involved in adaptive evolution and pathogen resistance have been identified in various amphibian species [1, 3, 10, 11]. In this study, *de novo* transcriptome sequencing of the skin of three amphibians, *A*. *davidianus*, *B*. *gargarizans*, and *R*. *Hallowell*, generated approximately 17 Gb of clean nucleotides for each species and identified a total of 167,064, 271,117, and 260,306 unigenes, respectively. These newly generated transcripts enrich the genome resource of amphibians and will facilitate biological research for the entire taxon.

Skin is the first barrier to protect amphibians against external invasions and plays essential roles for their survival on land. Despite a wide range of amphibian species, the functions of skin are largely conserved among this taxon, including respiration, immunity, and wound repair [7, 12, 18, 19]. In this study, *A*. *davidianus* belongs to the caudate group, whereas *B*. *gargarizans* and *R*. *Hallowell* are sister-groups of anurans. GO and KEGG classifications of unigenes indicated that skin functions were highly conserved across these three species. First, the GO terms related to basic functions, such as “metabolic process,” “binding,” “cell,” “cell part,” “catalytic activity,” and “cellular process,” were predominantly clustered for the three amphibians. This result is consistent with a previous study on transcriptome sequencing of skins of seven anurans [1], which emphasized the basic biological functions of amphibian skin. Additionally, many unigenes were in the GO terms “immune system process” (GO:0002376) and “response to stimulus” (GO:0050896) and in the KEGG pathways of “signal transduction” and “immune system,” suggesting that the skin plays important roles in the immune system of amphibians. Moreover, unigenes in pathways of “translation,” “transport and catabolism,” “folding, sorting and degradation,” and“ribosome” were highly represented in all three amphibian skins. Similar results are also reported in other amphibian skin transcriptome sequencing projects [1], which may be related to the generation of many mucus proteins and peptides in amphibian skins. The high proportion of immune-related genes expressed in amphibian skins suggests that immune function is one of the most important evolutionary adaptions of amphibian skin.

Amphibians are a large group that contains more than 6,800 species [16]. Different species show great variations in their phenotypes and life habits. Thus, extensive divergence in both molecular functions and structures likely occurred during the evolution of this taxon. In this study, 1,147 single-copy orthologs were used to evaluate the putative molecular basis underlying the differentiation of these three species. For each amphibian species, both the conserved and divergent orthologs were predominantly enriched in GO terms in the cellular component category. This result revealed that cellular component-related genes are important in maintaining the basic cell structure and function of amphibians and also that variation of these genes may cause large differences in their appearance and adaption. Moreover, a notable GO term distribution difference in the biological process category was identified for conserved orthologs between *A*. *davidianus* and the other two amphibians (*B*. *gargarizans* and *R*. *Hallowell*) (Fig 3B). The differently enriched GO terms were primarily related to carbohydrate metabolism and catabolic process (S3 Table). This is consistent with the difference in respiration function of skins of the three amphibians: *A*. *davidianus* is a lungless species and skin contributes to almost 70% of oxygen exchange, whereas oxygen exchange of *B*. *gargarizans* and *R*. *Hallowell* is less reliant on the skin [20]. This result provided evidence for skin functional differentiation across amphibians at the molecular level.

Despite the differences in structure and function, the innate immune functions of amphibian skins were commonly shared, particularly the immune function of anti-microbial defense. KEGG enrichment showed that pathways related to nucleotide metabolism and immune response were significantly clustered for conserved orthologs in all the amphibians (Table 3). The immune response pathway, “cytosolic DNA-sensing pathway” (ko04623), contains specific receptors that are responsible for detecting foreign invasion and generating innate immune responses (http://www.genome.jp/dbget-bin/www_bget?pathway:map04623). One gene in this pathway, DNA-dependent RNA polymerase III (Pol-III), was highly conserved across the three amphibians. RNA Pol-III is a cytosolic DNA sensor that plays the role of goalkeeper in protection against external invasions by converting the invasion DNA into RNA for recognition by the RNA sensor RIG-I [21]. These results indicated that amphibians retained common anti-microbial mechanisms during evolution, which played essential roles in their adaption and survival on land. Furthermore, several stimulus responses-related PSGs were identified in each of the three amphibians (S3 Table). This further emphasized the function of skin in innate immune systems of amphibians.

In addition to the internal anti-microbial biological processes, AMPs, which are secreted by amphibian skin, can form a natural immune defense to prevent bacterial infections [9, 22, 23]. Previous studies based on transcriptome sequencing successfully identified different types of AMPs in amphibian skins [1, 7, 10]. In this study, we identified a total of 50 AMPs from 26 different AMP categories in the three amphibian skins (Table 4). This result indicated that transcriptome sequencing is a reliable method to identify AMPs, which will be become more efficient as additional AMP sequences are published. Among the three species, *R*. *Hallowell* contained the highest the number of AMPs representing the most families, in addition to the highest levels of expression for most of the AMPs, which might explain the wider distribution of *R*. *Hallowell* than *A*. *davidianus* and *B*. *gargarizans*.

In conclusion, *de novo* transcriptome sequencing was conducted to explore the molecular basis underlying immune function of amphibian skins. Unigenes enriched in GO terms and KEGG pathways that were related to basic metabolism, cellular component, and immunity were most common among the three amphibians. Additionally, genes related to immune function were highly represented in both the conserved orthologs and PSGs in all the amphibians. Our study has increased understanding of the molecular basis for the immune functions of amphibian skin, and the transcriptome data set generated in this study will facilitate future molecular biology studies on amphibians.

## Materials and methods

### Ethics approval

All the methods involving animals in this study were performed in accordance with the Laboratory Animal Management Principles of China. The Ethics Committee of Chongqing University of Arts and Sciences approved all experiments.

### Sample collection and library construction

The Asiatic toads (*Bufo gargarizans*) and Heiban frogs (*Rana nigromaculata Hallowell*) were artificial raised that purchasing from the Gaohan Aquatic Animal Husbandry Company (Chongqing, China; Registered Number: 500240000271603; Lisence Number: Yu-2013-58). The Chinese giant salamanders (*Andrias davidianus*) were purchasing from the Chongqing Pengshuishunyu Rare Aquatic Animal Husbandry Company, which was supervised and permitted by the Chongqing fishing and fishing port supervision and management office. The permission of using rare animals was shown in S4 and S5 Figs. The *A*. *davidianus* used for sampling were four years old, and the *B*. *gargarizans* and *R*. *nigromaculata Hallowell* were two years old. The skins of *A*. *davidianus*, *B*. *gargarizans* and *R*. *nigromaculata Hallowell* were first cleaned and sterilized using 75% alcohol before sampling. The animals were released to aquatic farms after sampling. The surgery on all animals was performed under anesthesia using 500 mg/L MS-22, and approximately 1 square centimeter of skin tissue was cut from each individual. After removing the underlying tissues, the skins were frozen immediately in liquid nitrogen and stored at -80°C before RNA extraction. To eliminate individual differences in gene expression, skin from three individuals (both male and female) of each species was mixed for RNA isolation. Total RNA was extracted using Trizol reagent (Invitrogen, USA) according to the manufacturer’s instructions. Concentrations of RNA samples were detected by Qubit 3.0 (Thermo Scientific, UAS), which was followed by a quality assay using an Agilent 2100 Bioanalyzer (Agilent Technologies, USA). The strand-specific RNA-seq libraries were prepared using a NEBNext Ultra Directional RNA Library Prep Kit (cat#E7420; NEB, UK) according to the manufacturer’s instructions. Generated libraries were sequenced on a Hiseq 4000 (Illumina, San Diego, USA) platform using a paired-end run (2 × 150 bp).

### Transcriptome assembly and functional annotation

Raw reads for each library were first filtered to remove sequence adaptors and reads with quality under Q20. The generated clean reads were subjected to quality control using FastQC software before further analysis. Clean reads of *A*. *davidianus*, *B*. *gargarizans*, and *R*. *Hallowell* were *de novo* assembled into contigs using the program Trinity [24] with default parameters. The clean reads were re-mapped to these assembled contigs to calculate the coverage of contigs. Finally, the longest contig for each transcript was treated as a unigene in subsequent analyses. The expression levels of the unigenes for each species were evaluated by the RSEM 1.2.31 package [25] using the FPKM (fragments per kilobase of exon per million fragments mapped) method.

For functional annotation of unigenes, the CDS and protein sequences of each unigene were first predicted using TransDecoder (http://transdecoder.github.io/). Functional categories of the unigenes were determined using the BLASTX program searching against five gene databases, including the Cluster of Orthologous Groups of proteins (COG) database (https://www.ncbi.nlm.nih.gov/COG/), the NCBI non-redundant (NR) database (https://www.ncbi.nlm.nih.gov/refseq/about/nonredundantproteins/), the UniProt database (http://www.uniprot.org/), the Gene Ontology (GO) database (http://www.geneontology.org/), and the Kyoto Encyclopedia of Genes and Genomes (KEGG) database (http://www.genome.jp/tools/kaas/) (E-value threshold of 1 × 10^−5^).

### Phylogenetic and evolutionary analyses

For phylogenetic analysis, single-copy ortholog genes among the four species *A*. *davidianus*, *B*. *gargarizans*, *R*. *Hallowell*, and *Xenopus tropicalis* (*X*. *tropicalis*, used as the out-group species) were identified using OrthoMCL 2.0.3 software [26] from the assembled transcriptome or genome data. The genome sequence of *X*. *tropicalis* was downloaded from its genome data in bioMart (ftp://ftp.ensembl.org/pub/release-75/fasta/xenopus_tropicalis/). The phylogenetic tree was constructed using the concatenated sequences of all single-copy orthologs by FastTree software [27] with a maximum likelihood (ML) method and bootstrap replicates of 1,000.

For evolutionary analysis, the ratio of non-synonymous substitution (Ka) to synonymous substitution (Ks) of each single-copy ortholog was calculated using KaKs-Calculator software (version 2.0). Ka/Ks < 0.1 [28] was used to identify conserved orthologs, whereas evolutionarily divergent orthologs were identified by Ka/Ks > 1. For analyses of the functional significance of these conserved and divergent orthologs, GO and KEGG enrichment was performed to identify significantly enriched (false discovery rate, FDR < 0.05) GO terms and KEGG pathways.

### Positively selected genes (PSGs) estimation

The single-copy orthologs used for phylogenetic analysis were subjected to PSG identification using KaKs-Calculator software (version 2.0) with the MYN model [29]. Orthologs with a Ka/Ks value > 1 and a corrected *P*-value < 0.05[30] were inferred as PSGs.

### Putative antimicrobial peptide (AMP) identification

For putative AMP identification, all assembled unigenes were aligned against the databases of anuran defense peptides (DADP) (http://split4.pmfst.hr/dadp/) [31] and NR using the BLASTX program with E-value ≤ 1 × 10^−5^.

### Antimicrobial assay of an AMP

To validate the function of putative AMPs, a common AMP among the three amphibian species was randomly selected and synthesized (Qiangyao Bio-Tek, Shanghai, China) for use in an antimicrobial assay. The AMP was dissolved in acetic acid solution (30%) to a final concentration of 5,120 μg/mL and then sterilized using a 0.22 μm filter membrane before use. The standard bacterial strains *Salmonella*, *Escherichia coli* (*E*. *coli*), and *Staphylococcus aureus* (*S*. *aureus*) were purchased from XX Company (XX, China). Other strains that included *Chryseobacterium*, *Klebsiella pneumonia* (*K*. *pneumoniae*), *Aeromonas hydrophila* (*A*. *hydrophila*), *Shewanella*, and *Citrobacter* were isolated in our laboratory.

The minimum inhibitory concentration (MIC) was determined according to the standard method of the European Committee for Antimicrobial Susceptibility Testing [32]. Briefly, the AMP solution was serially diluted to concentrations of 512, 256, 128, 64, 32, 16, 8, 4, 2, and 1 μg/mL for the antimicrobial assay. One hundred microliters of respective AMP dilution was added to 100 μL of fresh overnight inoculum (1 × 10^5^ cfu/mL) and incubated at 37°C for 18–22 h. Finally, the absorbance at 600 nm of the cultures was detected to define the MIC for each bacteria.

## Supporting information

S1 Fig Length distributions of unigenes of each amphibian skin transcriptome. Bars with different colors represent different species.
(TIF)

S2 Fig The top 15 hit species for *A*. *davidianus* (A), *B*. *gargarizans* (B), and *R*. *Hallowell* (C) in the NR database based on unigenes. (TIF)

S3 Fig The KaKs distributions of single-copy orthologs in *A*. *davidianus* (A), *B*. *gargarizans* (B), and *R*. *Hallowell* (C). The KaKs values of orthologs in each species were calculated using *X*. *tropicalis* as reference. Red dots indicate conserved orthologs. Green dots indicate divergent orthologs. Blue dots indicate orthologs under neutral selection.
(TIF)

S4 Fig Permission of using rare animals (in English). (TIF)

S5 Fig Permission of using rare animals (in Chinese). (TIF)

S1 Table KEGG classification of all assembled unigenes from the three amphibian skin libraries. (XLS)

S2 Table GO classification of conserved (a) and divergent (b) orthologs in each amphibian. (XLS)

S3 Table Positively selected unigenes in *A*. *davidianus* (a), *B*. *gargarizans* (b), and *R*. *Hallowell* (c). (XLS)
