# Supplementary figures and images for: Comparative transcriptome analyses reveal the genetic basis underlying the immune function of three amphibians’ skin
Source: PLoS One. 2017 Dec 21;12(12):e0190023. doi: 10.1371/journal.pone.0190023 (PMC5739465; doi:10.1371/journal.pone.0190023)

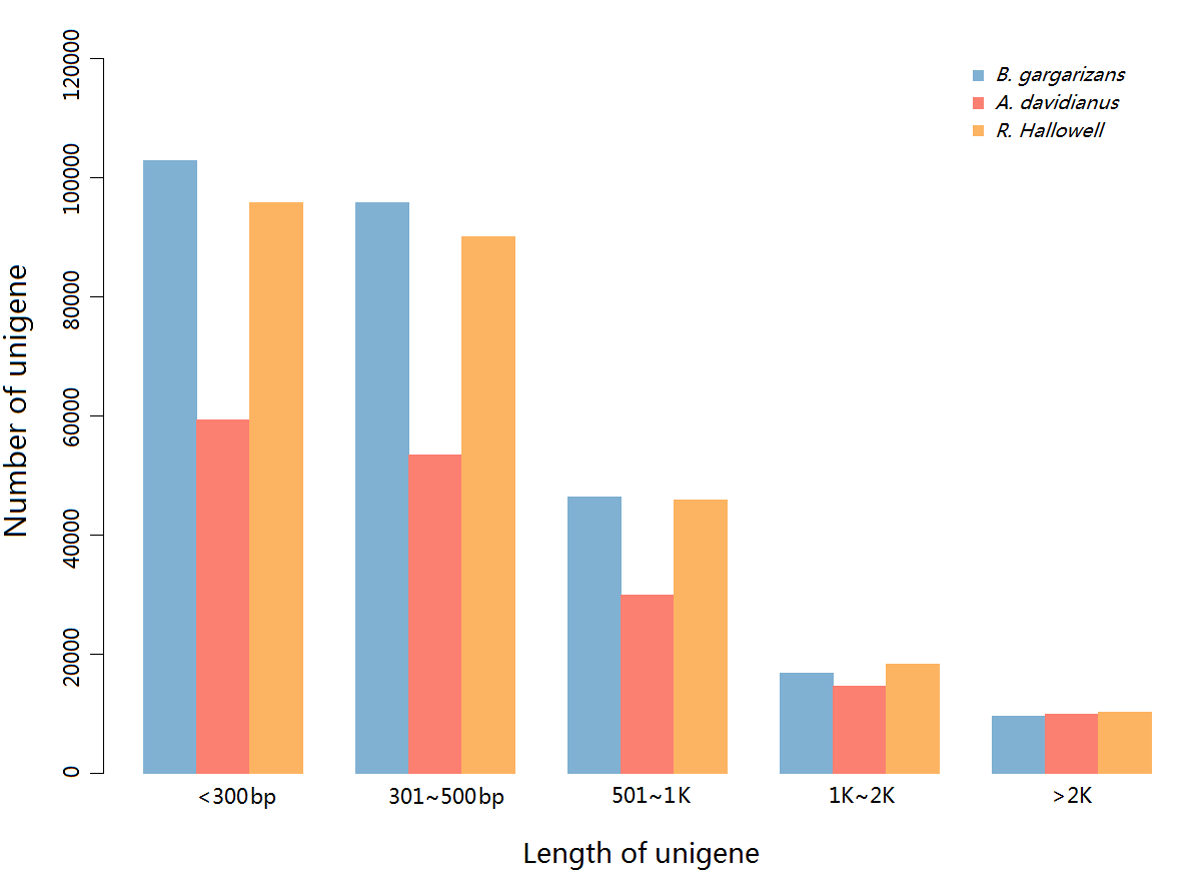

Supplement: S1 Fig — Bars with different colors represent different species. (TIF) [file pone.0190023.s001.tif]

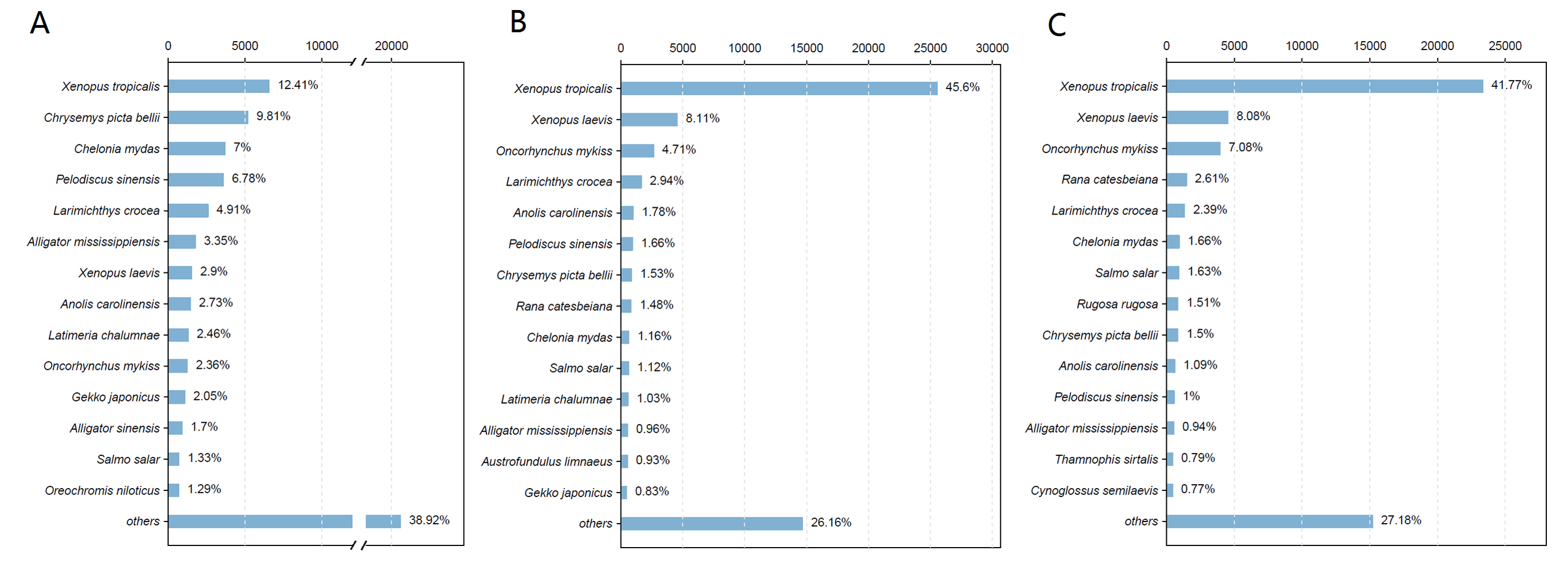

Supplement: S2 Fig — (TIF) [file pone.0190023.s002.tif]

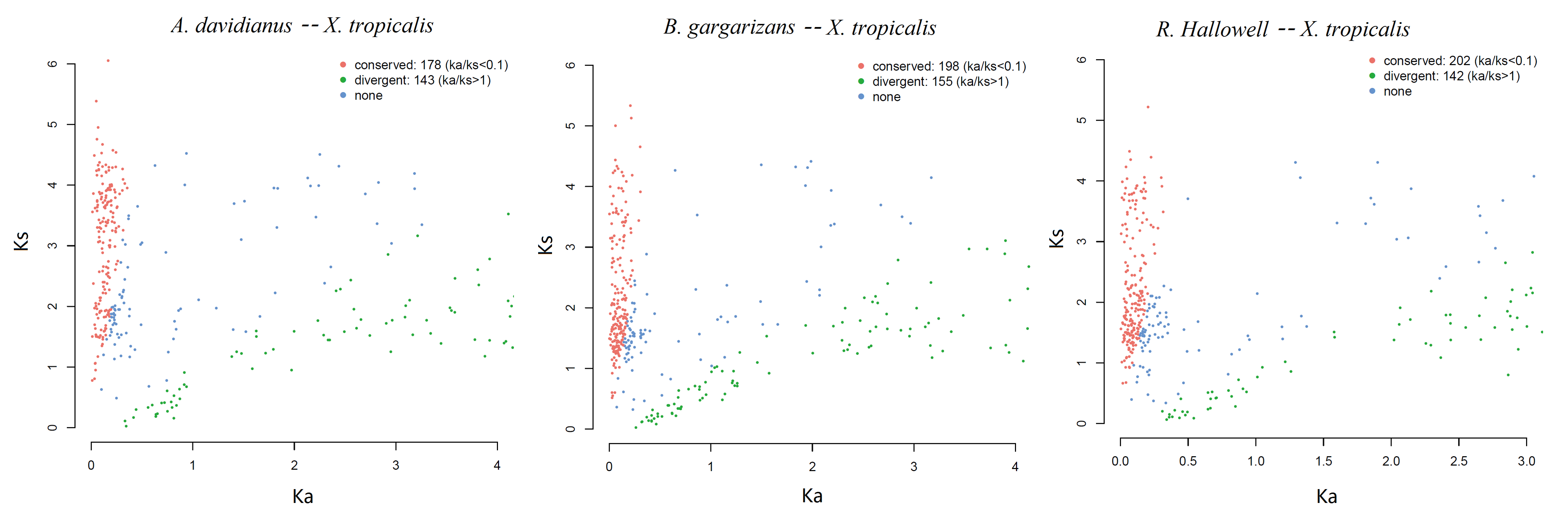

Supplement: S3 Fig — The KaKs values of orthologs in each species were calculated using X. tropicalis as reference. Red dots indicate conserved orthologs. Green dots indicate divergent orthologs. Blue dots indicate orthologs under neutral selection. (TIF) [file pone.0190023.s003.tif]

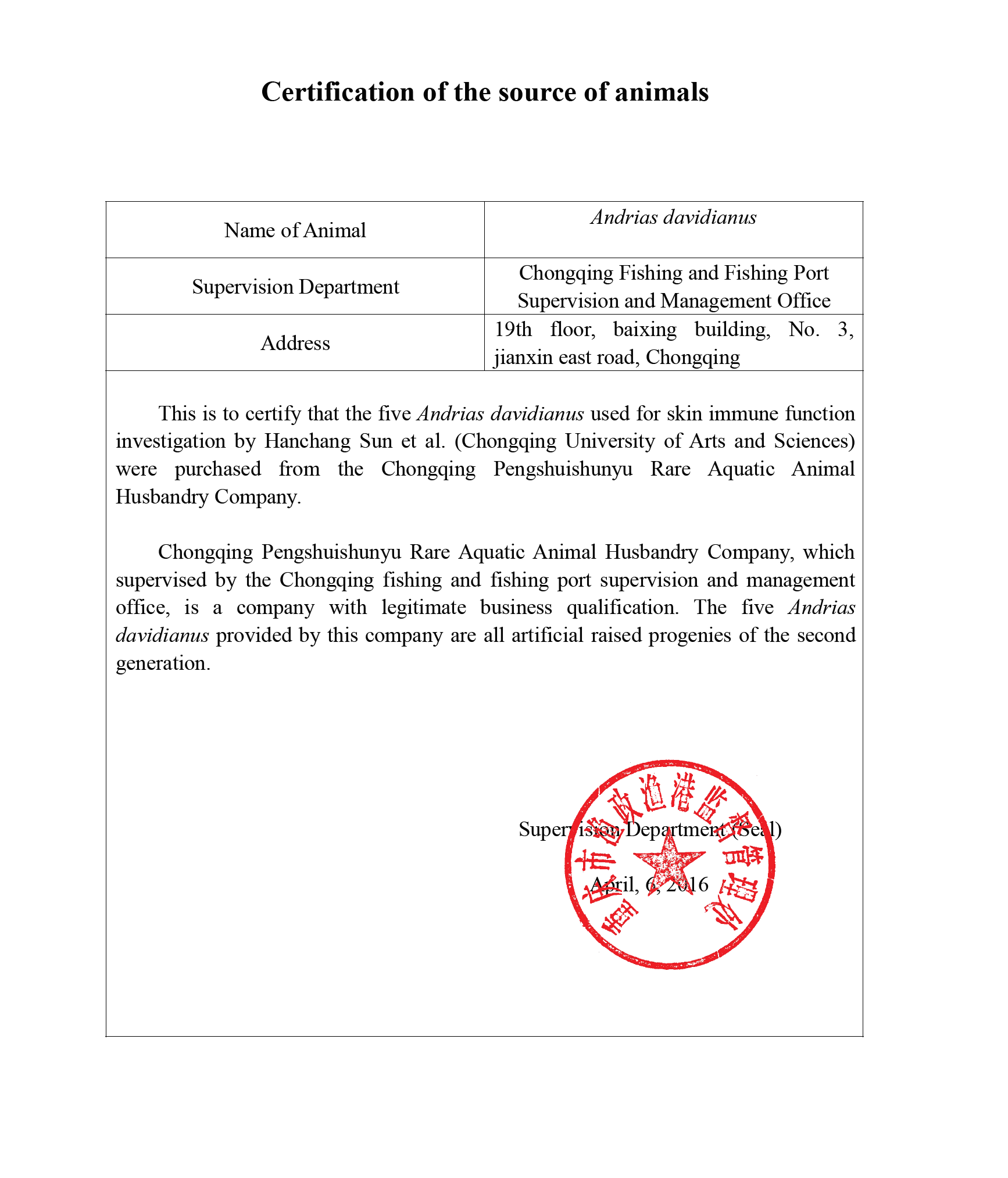

Supplement: S4 Fig — (TIF) [file pone.0190023.s004.tif]

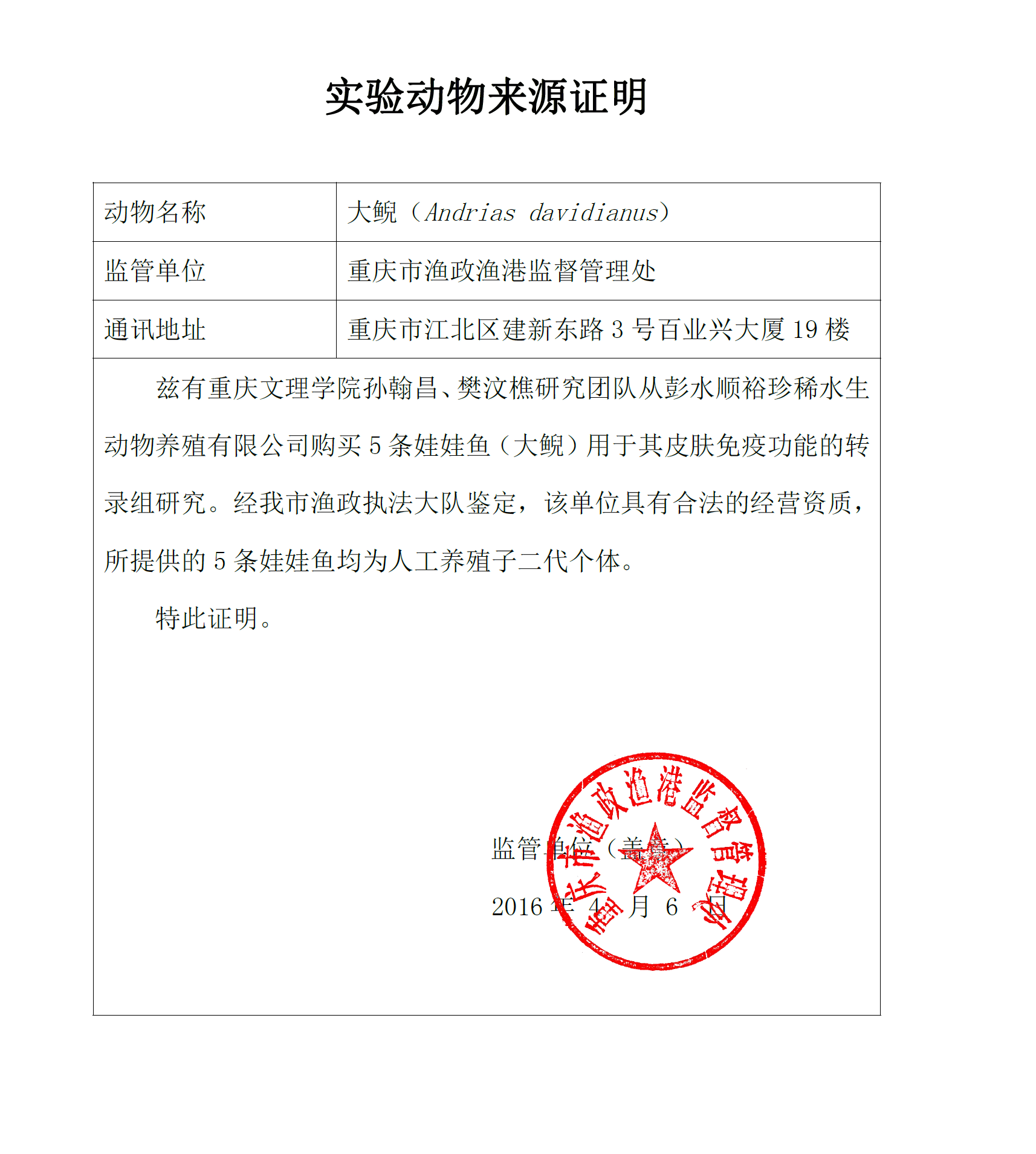

Supplement: S5 Fig — (TIF) [file pone.0190023.s005.tif]
